# Assessing the psychosocial work environment of migrant and non-migrant workers in inpatient mental health centres: A feasibility study

**DOI:** 10.1371/journal.pone.0275003

**Published:** 2022-09-28

**Authors:** Oriana Handtke, Lisa Viola Günther, Mike Mösko

**Affiliations:** 1 Department of Medical Psychology, University Medical Center Hamburg-Eppendorf, Hamburg, Germany; 2 Department of Applied Human Sciences, Magdeburg-Stendal University of Applied Sciences, Stendal, Germany; Post Graduate Institute of Medical Education and Research, INDIA

## Abstract

The number of migrant workers in Germany has increased over the last decades and will probably further increase in the context of a growing cultural diversity of the population and shortage of skilled professionals. Since migrant workers face different challenges, they may experience poorer psychosocial work environments than non-migrants. A negative psychosocial work environment can increase burnout and depression symptoms. To this date no study has investigated differences in the perceived psychosocial work environment in the mental health field. The aim of this study is to evaluate the feasibility of a cross sectional study comparing the perceived psychosocial work environment of migrants and non-migrant workers in inpatient mental health centres in Germany. The study was conducted in four inpatient mental health centres in Germany using the Copenhagen Psychosocial Questionnaire. All staff members (N = 659) categorized in seven professional groups were invited to participate in the study. The feasibility of the study was determined by four criteria (1) Implementation of the study in inpatient mental health centres (2) Representativity of the sample (3) Reliability and usability of the questionnaire and (4) Variability of collected data. Three of four feasibility criteria were achieved. The study was successfully implemented in four mental health centres, the usability of the used questionnaire was confirmed as well as the variability of the data. The targeted response rate was partially met, and the total number of migrant workers could not be provided, which limits the representativity of the sample. In conclusion, a main study is feasible, but an effort must be put in an effective recruitment strategy to obtain valid results.

## Introduction

Worldwide there are 164 million migrant workers [1]. 68% of migrant workers live in high income countries and Germany is after the United States the primary destination of international migrants [1]. In 2018, 18% of the workforce in Germany were foreign born. Including workers that were born in Germany from migrant descent, the proportion of migrant workers increases to 25% of the German working population [2]. The most common countries of origin are Turkey, Poland, Romania and Italy [3]. As in most European countries, migrant workers often work as low-wage and semi or unqualified workers [4]. Workers in the cleaning trade present the highest proportion of migrant workers with 33%, followed by food production and preparation with 31%. However, in the context of shortage of skilled professionals in Germany, the number of migrant workers increases also among qualified workers [3]. Especially, in the medical field, where physicians and nurses are needed, the number of migrant workers has been growing. In 2019, 7% of workers in the medical field were foreign-born [3]. The number of foreign-born physicians has doubled since 2011 and increased to 12% of working physicians in 2018 [5] while 15% of nurses in Germany are migrant workers [6].

Internationally, qualified and unqualified migrant workers appear to be particulary vulnerable to be confronted with problematic psychosocial work environments. Psychosocial work environment refers to “interpersonal and social interactions that influence behavior and development in the workplace” [7]. Theoretical models suggest that workers’ mental health is associated with risk (e.g. high demands, job insecurity) and protective (e.g. high social support, predictability) factors of the work environment [8–12]. Research confirmed this relationship and showed that high demands, low job control, high work load, low reward and job insecurity increase burnout and depression symptoms, while high levels of job support and workplace justice serve as protective factors [13–16]. Notably, migrant workers are more likely to work in high demand jobs, below their level of qualification, in low-skilled positions, in low security jobs and are exposed more often to harzadous working conditions than non-migrant workers [16–21]. Additonally, they face stress factors such as language barriers, cultural differences and discrimination [18,22–24]. However, research also presents evidence that migrant workers report better psychosocial work environment than non-migrant workers [25,26].

To this date, there is no study comparing perceived psychosocial work environment of migrant and non-migrant workers in the mental healthcare sector. Generally, little is known about the work environments in the mental health sector, but mental health workers have a particularly high risk of experiencing stress and burnout compared to workers in other sectors and professions [27,28]. A review of studies concentrating on burnout among applied psychologists notes that workload and perceived time pressure were identified as the most significant job demands. Among community-based mental health service providers, the work environment was characterized by unfavorable leadership style, role conflicts and work overload, which were predictive of burnout symptoms [28].

We plan a multicenter cross-sectional study to identify differences in perceived psychosocial work environment of migrants and non-migrants in inpatient mental health centres in Germany. However, getting access to inpatient mental health centres for research and involving health professionals in research can be challenging due to lack of time or interest in the research topic and doubts about its value [29–31]. Further, the proportion of migrants working in inpatient mental health centres is unknown and challenges in their recruitment can lead to their underrepresentation in study samples [32]. To ensure that the implementation of the study including recruitment, data collection and data interpretation will be successful, we decided to conduct this feasibility study. The aim of this study is to evaluate the feasibility of a cross sectional study comparing the perceived psychosocial work environment of migrants and non-migrants in inpatient mental health centres in Germany.

## Materials and methods

This study was funded by the German Social Accident Insurance Institution for the Health and Welfare Services and its ethical approval was obtained by Ethics Committee of The Chamber of Psychotherapists in Hamburg. Study participation was volontary and data collection was anonymous. Additionally, an information letter was attached to the questionnaire. This stated that by sending back the questionnaire the participants agree to their data being processed in this study. The names of the participating centres were not declared.

### Setting and participants

The study was conducted in cooperation with four inpatient mental health centres in Germany, which are referred to as Centre A, B, C and D. All centres offer inpatient rehabilitative mental healthcare, which is covered by the German pension insurance. Centre C is specialized on treating patients from Turkey or from Turkish descent and offer treatment in Turkish additionally to German. All staff members were eligible for participation. The total population of staff members was N = 659 with n = 151 in Centre A, n = 174 in Centre B, n = 144 in Centre C and n = 190 in Centre D. Staff members were categorized into administrative staff (n = 57; 8.6%), kitchen- and cleaning staff (n = 96; 14.6%), nurses (n = 136; 20.6%), physicians (n = 62, 9.4%), psychologists/psychotherapists (n = 110; 16.7%), therapists (occupational, physical, dance, music, dietitians, social workers) (n = 127; 19,3%) and others (n = 71; 10.8%). The proportion of migrant workers in each centre could not be provided by the centres’ leadership because the cultural background of employees was either not collected or was preserved by data protection policy.

### Objectives of the feasibility study

Feasibility was evaluated by measuring following aspects (1) Implementation of the study in inpatient mental health centres (2) Representativity of the sample (3) Reliability and usability of the questionnaire and (4) Variability of collected data [33].

Following criteria of feasibility were determined:

1. Each step of the implementation plan (flow diagram 1) needs to be achieved in each centre
2. A. Response rates ≥ 40% overall, in each centre and in each professional group based on the response rate in the German COPSOQ validation report [12].
  B. Unfortunately, the total number of migrant workers in the centres is unknown. Therefore, the response rate of migrant workers cannot be determined. The most common countries of origin are Turkey, Poland and Italy in the general working population in Germany [2]. Therefore, these should be the most common countries of origin in the sample. Migrant workers are defined as workers whose parents or themselves migrated to Germany [2].
3. A. Internal consistency of each scale should be acceptable with Cronbach’s Alpha ≥ 0.7 [34]
  B. Small amount of total missing value of < 5% [35]
  C. Feedback on the questionnaire
4. Mean differences of ≥ 3 points between migrant and non-migrant workers on ≥ 5 scales [36]. Differences of ≥3 points are interpreted as clinically relevant since a difference of 3 points generally represents a statistical difference of small effect size (Cohen’s d = 0.2) [36].

### Study implementation plan

**Fig 1:**
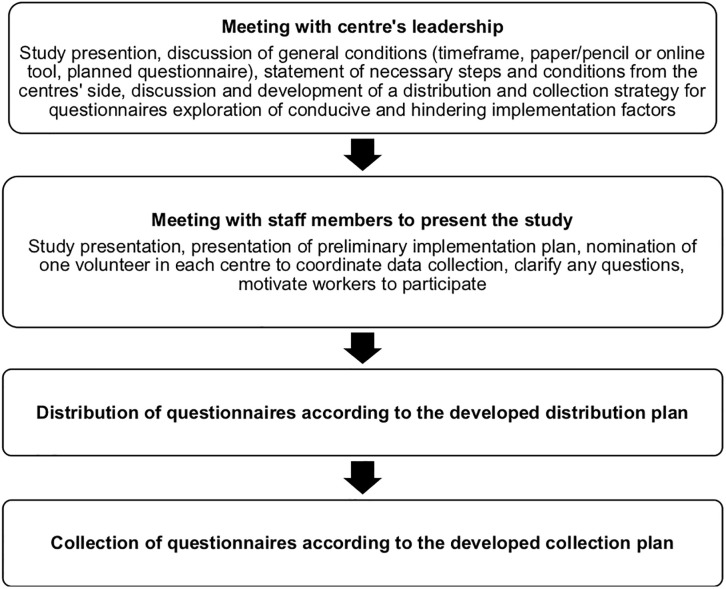
Study implementation plan.

A study implementation plan with necessary steps was developed a priori by the research team and is presented in Fig 1.

### The questionnaire

The validated Copenhagen Psychosocial questionnaire (COPSOQ) was used to assess psychosocial factors and work strain. The COPSOQ is a well-established tool used across different sectors. It is based on the Job-Demand-Support model [9] and the Effort-Reward Imbalance model [11] but aims to cover a broader concept of psychosocial stress and factors of the work environment [12].

The questionnaire consisted of 80 items attributed to 24 scales organized in five parameters (“Demands”, “Influence and development”, “Interpersonal relations and leadership”, “Further parameters” and “Outcomes”). The short version of the COPSOQ in German was used for the “Demands” section and its middle version for the sections “Influence and development“, “Interpersonal relations and leadership” and “Further parameters”. For economic reasons, further shortenings were made in the sections “Further parameters” and “Outcomes” as suggested by the study’s validation report [12].

Scales were answered on a four, five, seven or ten points Likert scale. They ranged numerically from 0 to 100 with, 0 representing the lowest anchor points and 100 the highest [37]. The answer “I have no supervisor/no colleagues” was treated as missing value, as instructed by the questionnaire’s validation report [12]. The COPSOQ presents good psychometric qualities (objectivity, reliability, validity, generalizability and sensitivity) with Cronbach’s Alpha between .60 and .90, except for the scale “Feedback (Cronbach’s Alpha = 0.58) [12,37,38].

The cultural background was assessed in the section “Statements about yourself and your workplace” at the end of the questionnaire. Participants were asked, if they or his/her parents were born in another country than Germany and if yes in which. Additionally, participants who migrated themselves were asked, for how many years they have been living in Germany.

In the section “Statements about yourself and your workplace” participants were also invited to indicate their sex, age, professional group, working time.

One open question at the end of the questionnaire asked for a feedback on the questionnaire.

According to the COPSOQ validation report it approximately takes 20 minutes to fill out the questionnaire.

### Methods of feasibility assessment

1. Feasibility of the study implementation plan was rated as successful if each step was implemented in all 4 centres. Methods and challenges in the implementation are reported.
2. A. Response rates were calculated overall, for each centre and each professional group.
  B. Percentage of migrant workers were calculated overall, in each centre and professional group. Also, their most common countries of origin were determined.
3. A. Percentage of missing value (<5%) was calculated.
  B. Cronbach’s Alpha was calculated for each scale and interpreted as α <0.7 (acceptable), α <0.8 (good), α <0.9 (very good) [34].
  C. Feedback given on the questionnaires was analyzed quantitatively and qualitatively with the qualitative content analysis [39].
4. Means and standard deviations of COPSOQ scales scores were calculated. According to recommendations from the validation report, scale scores were used to replace missing values if at least half of the single items on that scale were answered [12].

Data analysis was carried out with SPSS 28.

## Results

### (1) Feasibility of the study implementation plan

All steps of the implementation plan were successfully conducted in each centre. In the initial meetings with the centres’ leadership the study was briefly presented. All leaders recognized the importance of the topic and gave their consent for study implementation. The leader of Center A suggested that the distribution strategy should be discussed with all staff members to determine which is most convenient for them. This approach was then proposed to and approved by the other centres’ leaders. All leaders agreed that the timeframe to fill out the questionnaire should be 30 minutes and that staff members could answer it during worktime. Also, we agreed that the first reminder should be send 4 weeks after the first distribution if the response rate is not achieved. Since not all staff members have access to a personal computer at the centres, a paper/pencil survey was found more favorable than an online survey. Due to limited finances a small chocolate bar was included to the questionnaires as an incentive to be handed out to staff members. The leader of Centre B noted that a hindering factor for the study could be the small number of migrant workers. Unfortunately, the centres were unable to provide that exact number, because the cultural background of employees was either not collected or was preserved by data protection policy. This was an unexpected issue. Nonetheless, the lack of research on the given topic and the usage of a validated instrument were seen as conducive implementation factors by the centres. As all centres’ leaders pointed out, it was mandatory for the centre’s work council to check the study’s reasonableness and to approve the study. This step was unexpected, and it took on average 3 weeks for the work councils to approve the study. Only after the work councils’ approval the meetings with staff members could be organized. They were scheduled in the slots of the general staff meetings in each centre. Since general staff meetings do not include cleaning and kitchen staff and because of conflicting timetables, they were not present in the initial staff meetings, but their leaders were informed afterwards. In the staff meetings the theoretical background and the preliminary implementation plan were presented. A distribution and collection strategy for the questionnaires best suited for each centre was developed and a volunteer responsible for coordinating the study was found. Also, all questions of staff members were answered or discussed. Overall, staff members were interested and motivated to participate in the study. The primary concern was that they would not have time to fill out the questionnaire in their work time.

The questionnaire and an information letter about the study were distributed to all staff members either by the head of each professional group in their team meetings (Centre B), by putting them into the employees’ mailbox at the centre (Centre A and C), or by sending them with employees’ salary statements (Centre D). Three centres decided it was most convenient to collect the questionnaires in a designated box and to send the package to the researchers (Centre A, B, C). Centre D attached a return envelope, so employees could send the questionnaires directly back to the authors. As explained in the information letter participation was voluntary and anonymous and consent to participate was given by sending back the completed questionnaire.

Initial data collection took place in December 2016 in Centre A, in November 2017 in Centre B and C and in February 2018 in Centre D whith a small reminder via Email and/or in team meetings 4 weeks after the distribution of questionnaires. Initially, only one data collection phase was planned but due to small response rates staff members were aksed to participate again in January 2018 in Centre B and C as well as in April 2018 in Centre D.

The study was successfully implemented in each centre, but two additonal steps were necessary: getting the approval of the centre’s work council and a second data collection phase to improve response rates. Further, an important problem was identified, namley that the centres could not provide the total number of migrant workers.

### (2) Representativity of the sample

#### A. Response rates

The overall response rate was 37.5% (n = 247). More specific, the response rates were 49% (n = 74), 24% (n = 41%), 35% (n = 50) and 43% (n = 82) for Centre A, B, C and D respectively. As presented in Table 1. Response rates among professional groups were 54.4% (n = 31) for administrative staff, 24% (n = 23) for kitchen- and cleaning staff, 33.1% (n = 45) for nurses, 24.2% (n = 15) for physicians, 46.3% (n = 51) for psychologists/psychotherapists, 37.8% (n = 48) for therapists and 7% (n = 23) for others. The planned response rate of ≤40% was partially achieved: in centre A and D as well as for administrative staff and psychologists/psychotherapists.

**Table 1:** Sample characteristics stratified by migrants (N = 30) and non-migrants (N = 211).

Characteristics	Migrants (n = 30) n (%)	Non-migrants (n = 211) n (%)	Total (N = 241) n (%)
**Gender**			
Female	23 (77)	176 (83)	199 (83)
Male	6 (20)	30 (14)	36 (15)
Missing	1 (3)	5 (2)	6 (2)
**Age**			
≤ 24	1 (3)	7 (3)	8 (3)
25–34	10 (33)	35 (17)	45 (19)
35–44	8 (27)	51 (24)	59 (24)
45–54	3 (10)	81 (38)	84 (35)
≥ 55	7 (23)	35 (17)	42 (17)
Missing	1 (3)	2 (1)	3 (1)
**Working time**			
Part time (< 15 hours/week)	0	10 (5)	10 (4)
Part time (15–34 hours/week)	12 (40)	116 (55)	128 (53)
Full time (≥ 35 hours/week)	18 (60)	82 (39)	100 (4)
Missing	0	3 (1)	3 (1)
**Professional group**			
Administrative staff	4 (13)	27 (13)	31 (13)
Kitchen- and cleaning staff	4 (13)	19 (9)	23 (9)
Nurses	2 (7)	43 (20)	45 (19)
Physicians	4 (13)	11 (5)	15 (6)
Psychologists/Psychotherapists	9 (30)	42 (20)	51 (21)
Therapists	7 (23)	41 (19)	48 (20)
Others	0	23 (11)	23 (9)
Missing	0	5 (2)	5 (2)

#### B. Migrant workers

In six cases information about the cultural background was missing, so they were excluded from the analyses. The percentage of migrant workers was 12.4% (n = 30). 15 workers (50%) indicated they migrated themselves and 15 (50%) indicated their parents migrated to Germany. Table 1. presents sample characteristics. The most frequent countries of origin were Turkey (n = 7), Russia (n = 5), Italy (n = 2) and Poland (n = 2). Other countries of origin were Great Britain, Spain, Ukraine, Hungary, Slovakia, Morocco and Kyrgyzstan. Eight participants did not declare their country of origin. The most common country of origin was Turkey but also Poland and Italy, which was required by the feasability criteria.

The propotion of participating migrant workers were 8.1% (n = 6) in Centre A, 2.6% (n = 1) in Centre B, 24.5% (n = 12) in Centre C and 13,8% (n = 11) in Centre D. Among professional groups the proportion of participating migrant workers was 7.8% (n = 4) for administrative staff, 17.4% (n = 4) for kitchen and cleaning staff, 4.4% (n = 2) for nurses, 26.7% (n = 4) for physicians, 17.6% (n = 9) for psychologists/psychotherapists, 14.6% (n = 7) for therapists and 0% for others. Table 2 presents the numbers of participating migrant and non-migrant workers stratified by centre and professional group.

**Table 2:** Migrant and non-migrant workers stratified by centre and professional group.

	Centre A n = 74 (%)		Centre B n = 38 (%)		Centre C n = 49 (%)		Centre D n = 80 (%)		Total
	Mig.	Non-mig.	Mig.	Non-mig.	Mig.	Non-mig.	Mig.	Non-mig.	
Administrative staff	1 (1)	6 (8)	0	6 (16)	0	6 (12)	3 (4)	9 (11)	31 (13)
Kitchen- and cleaning staff	3 (4)	1 (1)	0	8 (21)	0	1 (2)	1 (1)	9 (11)	23 (10)
Nurses	0	18 (24)	0	1 (3)	1 (2)	8 (16)	1 (1)	16 (20)	45 (19)
Physicians	0	4 (5)	0	2 (5)	3 (6)	0	1 (1)	5 (6)	15 (6)
Psychologists/Psychotherapists	1 (1)	11 (15)	0	8 (21)	6 (12)	13 (27)	2 (3)	10 (12)	51 (21)
Therapists	1 (1)	15 (20)	1 (3)	11 (29)	2 (4)	2 (4)	3 (4)	13 (16)	48 (20)
Others	0	12 (16)	0	0	0	5 (10)	0	6 (8)	23 (10)
Missing	0	1 (1)	0	1 (3)	0	2 (4)	0	1 (1)	5 (2)
Total	6 (8)	68 (92)	1 (3)	37 (97)	12 (24)	37 (76)	11 (15)	69 (86)	241 (100)

### (3) Usability of the questionnaire

#### A. Reliability of the questionnaire

As a measure of the internal consistency of the COPSOQ scales Table 3 presents the number of items and Cronbach’s alpha of the COSOQ scales.

**Table 3:** Number of items and Cronbach’s alpha of the COSOQ scales.

Variables	Number of items	Cronbach’s Alpha
Demands		
Quantitative demands	3	.75
Emotional demands	2	.71
Hiding emotions	1	n.a.
Influence and development		
Influence at work	4	.69
Degree of freedom at work	4	.46
Possibilities for development	4	.82
Meaning of work	3	.89
Workplace commitment	4	.72
Interpersonal relations and leadership		
Predictability	2	.72
Role clarity	4	.83
Role conflicts	4	.81
Quality of leadership	4	.88
Social support	4	.70
Quality of feedback	2	.47
Social relations	2	- 1.19
Sense of community	3	.81
Further parameters		
Work-privacy conflict	5	.92
Mobbing	1	n.a.
Job insecurity	4	.76
Outcomes		
Intention to leave	1	n.a.
Job satisfaction	7	.80
General health	1	n.a.
Burnout	6	.88
Satisfaction with life	5	.88

The scales “Degree of freedom at work”, “Quality of feedback” and “Influence at work” were the only scales with Cronbach’s alpha <0.7 and therefore not acceptable. Cronbach’s alpha of the scale “Social relations” is negative, which means that the two items are negatively correlated and do not represent a reliable scale. Overall, the internal consistency of the scales is acceptable.

#### B. Missing value

The percentage of missing values of the COPSOQ was 1.9% and 3.5% of the sociodemographic questions. So, the criterion of missing value <5% was achieved.

#### C. Feedback on the questionnaire

Twenty-seven participants (11.2%) gave written feedback on the questionnaire. Nine comments approved or complimented the questionnaire as it is, thirteen comments proposed some improvements regarding the questionnaire or data collection, three comments criticized some aspects within the centres, one claimed that the questions were not easy to answer, and one gave an indication on the “relevance of the results for east and west Germany”. Following aspects were proposed to be added to the questionnaire: strain due to work underload, physical limitations, duties outside the area of responsibility or in replacement situations as well as private strain and appreciation of one’s work by colleagues and supervisors. It was further noted that it should be differentiated between “profession” and “job” as well as colleagues and supervisors, and that answers should also be asked in regard to the centre’s leadership not only supervisors. Also, it was indicated that in small institutions anonymity was not secured because questions were too personnel and that a secure return envelope should be provided to assure anonymity.

A generally acceptable internal consistency of the scales, a low percentage of missing values and positive comments on the questionnaire indicate a good a usability of the questionnaire. However, the elevated missing values of sociodemographic factors and the comments on anonymity indicate that participants have been worried about the confidentiality of their data.

### (4) Variability of the data

Table 4 shows means and standard deviations of both groups on each COPSOQ scale.

**Table 4:** COPSOQ scales’ means (standard deviations) and differences between migrant and non-migrant workers.

Variables	Migrant workers n = 31 M (SD)	Non-migrant workers n = 210 M (SD)
Demands		
Quantitative demands	56.3 (17.6)	55.8 (19.3)
Emotional demands	53.3 (21.3)	55.3 (22.0)
Hiding emotions	46.8 (28.0)	48.3 (27.8)
Influence and development		
Influence at work^1^	37.5 (20.2)	37.3 (19.3)
Degree of freedom at work^1^	45.0 (19.4)	45.8 (17.5)
Possibilities for development^1^	67.9 (14.9)	65.7 (19.8)
Meaning of work^1^	86.1 (14.2)	77.3 (19.6)*
Workplace commitment^1^	65.4 (16.8)	54.1 (18.9)*
Interpersonal relations and leadership		
Predictability^1^	57.1 (17.6)	52.6 (20.0)*
Role clarity^1^	77.9 (15.3)	72.5 (16.2)*
Role conflicts	43.7 (18.8)	43.6 (20.7)
Quality of leadership^1^	53.1 (24.4)	55.3 (23.7)
Social support^1^	65.6 (18.8)	67.4 (18.3)
Quality of feedback	51.3 (22.1)	42.8 (21.3)*
Social relations	52.5 (20.6)	49.7 (17.3)
Sense of community^1^	77.8 (17.7)	75.2 (18.2)
Further parameters		
Work-privacy conflict	45.7 (30.4)	35.8 (25.9)*
Mobbing	23.3 (24.1)	18.6 (24.4)*
Job insecurity	20.2 (18.8)	21.2 (19.0)
Outcomes		
Intention to leave	9.2 (16.7)	16.1 (21.0)*
Job satisfaction	71.2 (16.7)	67.6 (13.7)*
General health^1^	67.9 (16.1)	71.5 (18.7)*
Burnout	47.9 (20.5)	43.5 (17.5)*
Satisfaction with life^1^	67.4 (19.6)	68.9 (16.4)

^1^ High scores represent positive outcomes.

* Difference ≥ 3 points.

Differences between migrant and non-migrant workers of ≥ 3 points occurred on 11 scales: “Meaning of work”, “Workplace commitment”, “Predictability”, “Role clarity”, “Quality of feedback”, “Work-privacy conflict”, “Mobbing”, “Intention to leave”, “Job satisfaction”, “General health”, and “Burnout”. Therefore, the criterion of data variability is met, which supports the feasibility of the study.

## Discussion

This study evaluated the feasibility of a cross sectional study comparing the perceived psychosocial work environment of migrants and non-migrant workers in inpatient mental health centres in Germany. Three of four feasibility criteria were achieved. The study was successfully implemented in four mental health centres, the usability of the used questionnaire was confirmed as well as the variability of the data. The representativity of the sample is satisfactory but limited, since the targeted response rate was only partially met, and the total number of migrant workers could not be provided.

The study was successfully implemented in all four centres. The targeted response rate of 40% was achieved in Centre A and D as well as for administrative staff and psychologists/psychotherapists. The overall response rate (37.5%) and the response rate in Centre C (35%) and in the professional group of therapists (37.8%) came close to the targeted response rate. Other studies exploring the psychosocial work environment of health workers in hospitals reported response rates between 37% and 39.6% [8,40–42]. Studies using the COPSOQ questionnaire in geriatric care described response rates of 21% [26] and 33% [43] for nurses. Consequently, the feasibility goal was only partially met but the general response rate of 37.5% is similar to studies in the medical field. Generally, healthcare workers show low participation rates in survey studies due to time constraints, poor perceived value of the survey, problems with confidentiality, and perceptions of bias [44–46]. Worries about confidentiality were also expressed by participants in this study, especially migrant workers, because they are more easily identifiable. This could explain the elevated missing values on the sociodemographic questions. The low response rate of workers from the kitchen and cleaning staff could be explained by the fact that they were not present at the staff meeting where the study was present and did not know about the study or felt excluded. The topic of the questionnaire which is related to mental health may explain the good response rate of psychologists/psychotherapist who may have seen an important value of the study. This may have increased the motivation to participate [45]. Researchers have worked on strategies to improve response rates of healthcare workers, mainly focusing on physicians and nurses [47]. They are categorized into design- or incentive-based interventions [47]. A meta-analysis identified that mailed surveys, monetary compensation and one or two follow-ups produced the highest response rates [47]. In this study the highest response rate of Centre A (49%) was achieved without any follow-ups or monetary compensation, therefore other factors may have influenced the response rate. Possibly, (inter-)personal factors such as a good working climate, trust in the centres leadership and researchers regarding confidentiality or knowledge and perceived value of the study may have influenced the response rates [45,46,48]. Studies suggest that personalized contact from researchers, medical peers or authorities can also improve response rates [45,48]. Consequently, a focus should be put on engaging more personally with participants by personalized mail or a greater presence of the research team in the centres in the main trial. Also, an emphasis should be put on including kitchen- and cleaning staff in staff meetings and on recruiting physicians who had lower response rates than the other professional groups.

The distribution of migrant workers among different professional groups was unexpected compared to the general working population. The percentage of migrant workers in low wage and unqualified positions are high, especially in the cleaning trade or food production and preparation [3,4]. In this sample most migrant participants worked as qualified workers and were psychologists, therapists or physicians. The proportion of migrant workers among the kitchen and cleaning staff was with 5% (n = 1) lower as expected from the literature. The same is true for the sample of nurses, in which 4.4% (n = 2) were from migrant descent compared to 15% in the general population of nurses in Germany [6]. Nevertheless, the proportion of participating migrant physicians with 38.9% exceeded the number of migrant physicians (12%) in the population of working physicians in Germany [5]. The proportion of migrant workers among psychologists and physicians in this sample may have increased due to the focus on treating patients from Turkish descent of Centre C, which offers psychological and medical care in Turkish. Centre C had with 24.5% the highest rate of participating migrants, which conincides with the proportion of persons fom migrant descent in the general German population [3]. Accordingly, the most common country of origin was Turkey but also Poland and Italy, which also coincide with the general population of migrant workers in Germany [3].

Mean differences of perceived workplace environment of ≤3 points occurred on eleven scales, which according to the authors of the COPSOQ indicates a difference of small effect size [36]. A previous study found statistically significant differences with small to medium effect sizes between migrant and non-migrant nurses on the scales “emotional demands”, “work-privacy conflict”, “role conflicts” and “possibilities for development”. Mean differences were of ≤8 points. Mean differences of ≤3 points occurred on the scales “role clarity” and “sense of community”, but they were not statistically significant [26]. Similar to existing studies [25,26], this study found that migrant workers perceive their workplace environment equally or more favorable than non-migrant workers on seven of the eleven scales, but worse on the scales “work-privacy-conflict”, “mobbing”, “general health” and “burnout”. Importantly, since this is a feasibility study these results are not statistically significant but provide information on the variability of the data. The significance of these differences will be examined in the main trial.

The internal consistency of three scales were not acceptable. In comparison, the German COPSOQ validation study also reported Cronbach’s alpha <0.7 for the scales “Quality of feedback”, “Influence at work” and for “Social relations” [12]. The internal consistency of the scale “Degree of freedom at work” was good in the German validation study but insufficient in the Danish validation study [12]. Further, the scales “Quantitative demands” and “Job insecurity” did not achieve an acceptable internal consistency in the German validation study but did in this study [12]. Accordingly, the internal consistency in this feasibility study is comparable to the validation reports which supports the usability of the questionnaire in the target sample.

### Limitations

The feasibility study was only conducted in four mental health clinics. In other centres the implementation plan might not have been possible or other obstacles might have occurred.

An important and unexpected limitation was that the centres were not able to provide the total number of migrant workers. The proportion of participants form migrant descent in this sample was 12.4%. This has led to two different sample sizes (211 vs. 30) which can cause a loss of statistical power and small effects. In other studies, using the COPSOQ a difference in sample sizes were occurring but it was smaller or even negligible [25,26]. It is possible that the number of migrant workers were low in the included centres. A strategy to overcome this problem in the main trial is to ask in initial meetings with the centres’ leadership if this number is available and accessible for the researchers. If not or it the number is small, the centre is not eligible for study participation. Nonetheless, this is an important result of this feasibility study and identified a crucial problem in determining migrant workers response rate and therefore the representativity of the sample.

The targeted response rate of ≤40% which is based on the response rate of the COPSOQ validation report may be a limitation of this study. Compared to other research fields which target response rates of 60–70% it seems low which can impact the generalizability and applicability of the results [49]. Nonetheless, compared to studies from the same research field it appeared realistic [8,40–42].

To include second generation migrants in the group of migrant workers may be a limitation because they were professionally socialized in Germany. Nevertheless, workers from migrant descent may perceive their psychosocial work environment differently because they may be in charge of providing care for patients from migrant descent or offer treatment in different languages which can increase job strain [50]. If possible, it would be interesting to compare first and second-generation migrant workers in the main trial.

The questionnaire was only distributed in German and in written format which may have excluded migrant workers with limited German language skills. Indeed, their perceived psychosocial work environment may differ and be more challenging due to language barriers, so their answers may have been enriching.

## Conclusion

The study was successfully implemented in four mental health centres, the usability of the used questionnaire was confirmed as well as the variability of the data. The targeted response rate was partially met, and the total number of migrant workers rests unknown, which limits the representativity of the sample. In conclusion, a main study is possible, but the focus must be put on an effective recruitment strategy to obtain valid results.
